# Intra-individual variability in the neuroprotective and promyelinating properties of conditioned culture medium obtained from human adipose mesenchymal stromal cells

**DOI:** 10.1186/s13287-023-03344-1

**Published:** 2023-05-11

**Authors:** Vito Antonio Baldassarro, Francesca Perut, Maura Cescatti, Valentina Pinto, Nicola Fazio, Giuseppe Alastra, Valentina Parziale, Alessandra Bassotti, Mercedes Fernandez, Luciana Giardino, Nicola Baldini, Laura Calzà

**Affiliations:** 1 Department of Veterinary Medical Sciences (DIMEVET), University of Bologna, Via Tolara Di Sopra 50, 40064 Ozzano Dell’Emilia, Bologna, Italy; 2 Health Science and Technologies, Interdepartmental Center for Industrial Research (HST-ICIR), University of Bologna, Via Tolara Di Sopra 50, 40064 Ozzano Dell’Emilia, Bologna, Italy; 3 Biomedical Science and Technologies and Nanobiotechnology Laboratory, IRCCS Istituto Ortopedico Rizzoli, Via Di Barbiano 1/10, 40136 Bologna, Italy; 4 IRET Foundation, Via Tolara Di Sopra 41/E, 40064 Ozzano Dell’Emilia, Bologna, Italy; 5 Division of Plastic Surgery, Department of Medical and Surgical Sciences, University of Modena and Reggio Emilia, Via del Pozzo 71, 41124 Modena, Italy; 6 Department of Biomedical and Neuromotor Sciences, University of Bologna, Via Di Barbiano 1/10, 40136 Bologna, Italy; 7 Pharmacology and Biotecnology Department (FaBiT), University of Bologna, Via San Donato, 15, 40127 Bologna, Italy; 8 Monetecatone Rehabilitation Institute (MRI), Via Montecatone, 37, 40026 Imola, Bologna, Italy; 9 Present Address: Department of Chemical and Pharmaceutical Sciences, University of Ferrara, Via Luigi Borsari 46, 44121 Ferrara, Italy

**Keywords:** Mesenchymal stromal cells, Secretome, Neuroprotection, Remyelination, Growth factors

## Abstract

**Background:**

Greater knowledge of mesenchymal stromal cell (MSC)-based therapies is driving the research into their secretome, identified as the main element responsible for their therapeutic effects. The aim of this study is to characterize the individual variability of the secretome of adipose tissue-derived MSCs (adMSCs) with regard to potential therapeutical applications in neurology.

**Methods:**

adMSCs were isolated from the intact adipose tissue of ten subjects undergoing abdominal plastic surgery or reduction mammoplasty. Two commercial lines were also included. We analyzed the expansion rate, production, and secretion of growth factors of interest for neurological applications (VEGF-A, BDNF, PDGF-AA and AA/BB, HGF, NGF, FGF-21, GDNF, IGF-I, IGF-II, EGF and FGF-2). To correlate these characteristics with the biological effects on the cellular targets, we used individual media conditioned with adMSCs from the various donors on primary cultures of neurons/astrocytes and oligodendrocyte precursor cells (OPCs) exposed to noxious stimuli (oxygen–glucose deprivation, OGD) to evaluate their protective and promyelinating properties, using MSC medium as a control group.

**Results:**

The MSC secretome showed significant individual variability within the considered population with regard to PDGF-AA, PDGF-AB/BB, VEGF-A and BDNF. None of the MSC-derived supernatants affected neuron viability in normoxia, while substantial protection by high BDNF-containing conditioned MSC medium was observed in neuronal cultures exposed to OGD conditions. In OPC cultures, the MSC-derived supernatants protected cells from OGD-induced cell death, also increasing the differentiation in mature oligodendrocytes. Neuroprotection showed a positive correlation with VEGF-A, BDNF and PDGF-AA concentrations in the culture supernatants, and an inverse correlation with HGF, while OPC differentiation following OGD was positively correlated to PDGF-AA concentration.

**Conclusions:**

Despite the limited number of adMSC donors, this study showed significant individual variability in the biological properties of interest for neurological applications for adMSC secretome, an under-researched aspect which may represent an important step in the translation of MSC-derived acellular products to clinical practice. We also showed the potential protection capability of MSC conditioned medium on neuronal and oligodendroglial lineages exposed to oxygen–glucose deprivation. These effects are directly correlated to the concentration of specific growth factors, and indicate that the remyelination should be included as a primary target in MSC-based therapies.

**Supplementary Information:**

The online version contains supplementary material available at 10.1186/s13287-023-03344-1.

## Background

Cell therapies based on mesenchymal stromal cells (MSCs) and MSC-derived advanced therapy medicinal products (ATMPs) as extracellular vesicle or conditioned culture media have been the focus of increasing attention over the last decades as disease-modifying and/or disease-curing therapies and for regenerative medicine, and have been used in an impressive number of preclinical studies in almost all available disease models [1, 2]. This broad interest in MSC-based cell therapies runs in parallel with the increased knowledge on the biological properties of these cells, such as the trophic and immunomodulatory properties related to growth factors, regulatory molecules, and miRNA secretion [3]. The relative ease of sourcing these cells, together with their relatively low immunogenicity means that the prospect of their translation to clinical use in humans either as an autologous or allogenic transplant is high, as demonstrated by more than 1500 clinical trials registered at clinicaltrials.gov (keyword search: mesenchymal cells, May 2022), and more than 160 for neurological conditions [4, 5].

It is now widely recognized that therapeutic effect and possible regenerative potential of MSCs lies primarily in their secretome, which contains several growth factors, including specific pro-angiogenic and neurotrophic factors [6, 7]. MSC-conditioned culture medium offers a rich source of functional factors which have shown promising results in the treatment of various conditions and diseases such as limb ischemia, skin wounds, liver failure, kidney disease, alopecia, uveitis and bone defects [8–10].

The usually irreversible nature of neurological impairment due to the limited regenerative capability of the central nervous system (CNS) means that neurological lesions and diseases such as acute lesions of different etiologies, chronic degenerative diseases and neurodevelopmental disorders represent a preferential therapeutic area for MSC-based cell therapies, as shown by the impressive number of experimental studies. Neurological conditions such as stroke, traumatic brain injury, spinal cord injury, multiple sclerosis, amyotrophic lateral sclerosis, Alzheimer's disease, Huntington's disease and Parkinson's disease, characterized by inflammation and white matter damage, either in the form of primary and extended damage (stroke, traumatic brain injury, spinal cord injury, multiple sclerosis [11]), or as limited but clinically significant damage (amyotrophic lateral sclerosis, Alzheimer's disease, Huntington's disease, Parkinson's disease [12]), are considered prime candidates for MSC-based therapies [5]. Experimental studies suggest common mechanisms in these conditions, such as anti-inflammatory activity and the reduction of cell death, oligodendrogenesis and remyelination stimulation, scar reduction and the stimulation of angiogenesis.

The translation of experimental data to the clinical setting, however, is still thwarted by a lack of definitive data, with numerous aspects such as disease etiology, acute versus chronic conditions, therapeutic window, and delivery strategies remaining unclear. The advantages and disadvantages of autologous versus allogenic MSCs in medical practice are also still a subject of debate [13].

Research into MSCs has paid relatively little attention to the properties of donor cells, despite the fact that donor-related factors such as sex, age, systemic and autoimmune diseases, obesity and body mass index affect the quality, quantity and biological characteristics of MSCs from different body districts [14]. Even less consideration has been given to inter-individual variability in a homogenous donor population within the framework of clinical studies and the associated inclusion and exclusion criteria.

To address this shortcoming, we investigated various biological properties of human adipose-derived mesenchymal stem cells (adMSCs), focusing on individual variability of growth and neurotropic factors secretion and on the biological effects on neurons and oligodendroglial precursor cells (OPCs). In tissue taken from different sources, adMSCs showed a number of advantages, such as ease of harvesting using minimally invasive techniques, long-term phenotype maintenance, and low immunogenicity [15]. adMSCs also undergo senescence later than bone marrow-derived MSCs [16]. We collected MSCs from the adipose tissue of ten subjects undergoing abdominal plastic surgery or reduction mammoplasty, and analyzed the expansion rate, production, and secretion of growth factors of interest for neurological applications (VEGF, BDNF, PDGF-AA and AA/BB, HGF, NGF, FGF-21, GDNF, IGF-I, IGF-II, EGF and FGF-2). Two cell batches obtained from commercial providers (“COMM1” from ATCC and “COMM2” from LONZA) were also included in the study. To investigate the possible correlation of these biological characteristics with biological effects on potential cellular targets, we used individual adMSC-conditioned media on neurons and OPCs exposed to noxious stimuli to evaluate its protective and promyelinating properties. The neurons and OPCs were taken from the cerebral cortex and neural stem cells, respectively, then cultured and treated according to standardized protocols for drug discovery and testing [17, 18].

## Methods

### adMSC isolation and characterization

MSCs were isolated from the intact adipose tissue of ten subjects undergoing abdominal plastic surgery or reduction mammoplasty. The study was conducted in accordance with the Declaration of Helsinki, and the protocol was approved by the local research ethics committee (Comitato Etico di Area Vasta Centro della Regione Emilia-Romagna; authorization number: 152 354-2018-Sper-IOR—20th June 2018). Informed consent to use the discarded surgical tissue for research purposes was signed by all the enrolled patients. Exclusion criteria were the presence of benign or malignant neoplasms, diabetes, alcohol/drug abuse, and the presence of necrotic adipose tissue, and a code number was assigned to each sample to assure subject anonymity. Donor characteristics are described in Table 1. Medical history of donors enrolled in this study did not register any case of allergy, comorbidity or concomitant drug treatment. Only donor corresponding to ID sample 6006 assumed sodium levothyroxine (75 μg/day), due to hypothyroidism. The adipose tissue (~ 50 mL) was washed with sterile phosphate-buffered saline (PBS) several times and cut into small pieces. Fibrous tissue and blood clots were discarded. Enzymatic digestion was obtained with 0.075% type II collagenase (Gibco, Invitrogen, Monza, Italy) for 40 min at 37 °C under continuous shaking. To inactivate collagenase activity, low glucose Dulbecco's Modified Eagle Medium (DMEM) (Sigma-Aldrich, Milan, Italy) supplemented with 10% fetal bovine serum (FBS) (Sigma-Aldrich), was added at equal volume. Mature adipocytes and connective tissue were separated by centrifugation (400 g for 15 min) and filtered using a 100-μm mesh (Sigma-Aldrich). Freshly isolated stromal vascular fraction (SVF) was seeded (4.0 × 10^5^ SVF cells/cm^2^) and maintained in DMEM low glucose (Sigma-Aldrich) supplemented with 10% FBS, 100 U/ml penicillin and 0.1 mg/ml streptomycin (complete medium) at 37 °C in 5% CO_2_. The culture medium was discarded and replaced with fresh medium every 3–4 days.

**Table 1:** Demographic characteristics of donors and origin of tissue samples (type of surgery)

Sample code	Type of surgery	Age range	Sex	Height (meters)	Weight (kg)	BMI (kg/m^2^)
5898	Mammoplasty	60–69	F	1.85	130	37.98
5903	Abdominoplasty	20–29	M	1.67	80	28.69
5919	Mammoplasty	30–39	F	1.70	87	30.10
5959	Mammoplasty	30–39	F	1.70	85	29.41
5982	Mammoplasty	30–39	F	1.65	90	33.06
6006	Mammoplasty	40–49	F	1.65	73	26.81
6008	Abdominoplasty	20–29	F	1.61	51	19.68
6038	Mammoplasty	40–49	F	1.57	62	25.15
6111	Abdominoplasty	40–49	F	1.60	77	30.08
6113	Abdominoplasty	30–39	F	1.58	56	22.43

### adMSC immunophenotypic characterization

The immunophenotypic characterization of adipose-derived mesenchymal stromal cells (adMSCs) was assessed by direct immunofluorescence with flow cytometry analysis, labelling the adMSCs with fluorescin isothiocynate (FITC), R-Phycoerythrin (RD1) or RD1-Cyanin 5.1 (PC5) (Instrumentation Laboratory, Milan, Italy) monoclonal antibodies (CD14, CD34, CD44, CD45, CD90, CD105, CD73, CD106, CD160, HLA-DR). In brief, the cells (10^5^/test) were incubated for 20 min at 4 °C, washed and resuspended in PBS. The portion of positive cells was evaluated on 10,000 events/sample using a flow cytometer EPICS XL-MCL (Beckman Coulter, Fullerton, CA, USA).

### Osteogenic and adipogenic differentiation

For osteogenic differentiation, adMSC were seeded in 6-well plates at a density of 1 × 10^3^ cells/well and cultured until subconfluency. Then, the culture medium was replaced with medium consisting of *α*-MEM, 10% FBS, supplemented with 50 μg/mL l-ascorbic acid 2-phosphate (Sigma), 10^–8^ M dexamethasone (Sigma) and 10 mM *β*-glycerophosphate (Sigma) in order to induce the deposition of mineralized matrix. After 21 days, cells were fixed in 3.7% paraformaldehyde for 20 min and stained with 1% Alizarin Red (pH 4.2) (Sigma) for one hour at room temperature.

For adipogenic differentiation, adMSC were seeded in 6-well plates at a density of 1 × 10^3^ cells/well and cultured until subconfluency. Then the culture medium was replaced with complete medium supplemented with dexamethasone (0.5 μM), indomethacin (50 μM) (Sigma) and isobutylmethylxanthine (0.5 mM) (Sigma). After 14 days, to detect lipid accumulation, cultures were fixed in 3.7% paraformaldehyde for 20 min and stained with 0.3% Oil Red O in isopropanol for one hour at room temperature.

### Commercial cells

The adMSCs obtained from ATCC (PCS-500-011; lot: 60,866,843), referred as COMM1 throughout the text were isolated from a sample taken from a 38-year-old female subject during a liposuction procedure, while the cells obtained from LONZA (PT-2501, lot: 6F4085), referred as COMM2, were isolated by the Cambrex Bio Science Walkersville Inc. company from a 33-year-old male subject. The cells were characterized for MSC markers (positive for CD29, CD44, CD73, CD90, CD105, CD166; negative for CD14, CD31, CD34, CD45) and for their potential for differentiation into adipocytes, chondrocytes, and osteocytes.

### Conditioned media collection

Human adMSCs (1.0 × 10^4^ cells/cm^2^) from passage 2 to 3 were seeded on tissue culture flasks (25 cm^2^) in complete medium from passage two to passage seven. After 24 h, the cells were washed with PBS, and the medium replaced with low glucose DMEM supplemented with 5% FBS. To evaluate the specific effect of acidic pH on conditioned media activity, the adMSCs were also treated with low glucose DMEM (pH 6.8) supplemented with 5% FBS. The specific pH of the culture medium (6.8 or 7.4) was maintained using different concentrations of sodium bicarbonate, according to the Henderson–Hasselbach equation. Cells were maintained for 72 h at 37 °C in 5% CO_2_. At the end-point (72 h), the final pH of the conditioned media was measured with a digital pH-meter (6230N, Jenco, San Diego, CA, USA) in each case to ascertain the maintenance of the pH value throughout the incubation time. At the end of the incubation period, the adMSC-conditioned media was collected, and cell viability assessed using the erythrosine B (Sigma-Aldrich) dye exclusion method (Krause et al., 1984 PMID: 6,090,533). After centrifugation (1200 rpm for 5 min) to remove floating cells and cellular debris, aliquots of conditioned media, were prepared and stored at − 80 °C. The absence of mycoplasma contamination was evaluated by PCR (Mycoplasma Test Kit I/C) (Promocell, Heidelberg, Germany), and bacterial contamination evaluated by plate count agar (Technoanalyses Lab, Bologna, Italy).

### Determination of growth factors in adMSC-conditioned media

The growth factors VEGF-A, BDNF, PDGF-AA, PDGF-AB/BB, GDNF, HGF, FGF-21, IGF-I, IGF-II, NGF, EGF and FGF-2 were measured in the adMSC media conditioned for 72 h. The quantification was performed using a multiparametric assay based on Luminex xMAP technology and the Luminex 200 platform. Seven magnetic bead panels purchased from Merck-Millipore were used for the measurements: Human Angiogenesis Growth Factor Magnetic Bead Panel 1 (HAGP1MAG-12 K) to quantify VEGF-A; Human Neurodegenerative Disease Magnetic Bead Panel 3 (HNDG3MAG-36 K) to quantify BDNF, PDGF-AA and PDGF-AB/BB; Human Neurodegenerative Disease Magnetic Bead Panel 4 (HNDG4MAG-36 K) to quantify GDNF; Human Liver Protein Magnetic Bead Panel (HLPPMAG-57 K) to quantify HGF and FGF-21; Human IGF-I, II Magnetic Bead Panel (HIGFMAG-52 K) to quantify IGF-I and IGF-II, Human Adipokine Magnetic Bead Panel 2 (HADK2MAG-61 K) to quantify NGF and Human Cytokine/Chemokine/Growth Factor Panel A (HCYTA-60 K) to quantify EFG and FGF-2. The growth factor concentration values were obtained by interpoling the fluorescence data to specific standard analyte curves, using the xPONENT 3.1 software. Conditioned medium without cells was added to standard curves to exclude the matrix effect on measured values. Each media was processed in duplicate. Data are expressed as mean ± SEM.

### Primary cortical neurons

All protocols described herein were carried out according to the European Community Council Directives (86/609/EEC) and comply with the guidelines published in the *NIH Guide for the Care and Use of Laboratory Animals*. All the experiments were authorized by the Animal Welfare Body (AWB) of IRET Foundation (n. ID24-19,072,019—19th July 2019).

Cortical neurons from fetal mice (E13.5), isolated from a pregnant C57BL/6 (Charles River Laboratories Italia, Sant’Angelo Lodigiano, LO, Italy) were prepared according to a standard protocol [19]. Four animals were used to produce the primary cortical neuron experiments for the study. In brief, the pregnant mouse was sacrificed by cervical dislocation and embryos were extracted in a sterile Petri dish containing cold PBS 1 × with 100 U/ml/100 μg/ml Penicillin/Streptomycin. The brains were initially removed from the animals. The cortical tissue was then dissected, separated from the meninges, cut into small pieces, and dissociated in non-enzymatic dissociation buffer (Sigma-Aldrich) by mechanical pipetting following 15 min of incubation at 37 °C. Following centrifugation (500 × g, 5 min), the cells were resuspended in Neurobasal culture medium supplemented with 2% B27 (Gibco), 2 mM glutamine (Sigma-Aldrich), 100 U/ml penicillin, and 100 μg/ml streptomycin (Gibco), and plated onto Cultrex 2D substrate (0.25 mg/ml, Trevingen)-coated 96-well plates at 100,000 cells/well. The cells were maintained in a humidified incubator at 37 °C with 5% CO_2_, then treated with 10 μM cytosine arabinofuranoside (Ara-C; Sigma-Aldrich) after 24 h to obtain the neuronal culture (99% neurons). No mitotic inhibitor was used for mixed cultures. At 4 DIV, half of the medium was changed in both cultures.

### Neural stem cell-derived oligodendrocyte precursor cell isolation and cultures

Animals were purchased and prepared as described above. Four animals were used to produce the neural stem cell (NSC) experiments for the study. The OPCs were differentiated from NSCs isolated from the fetal forebrain, as already described [18]. In brief, the embryos were collected from pregnant female mice (E13.5) and placed in a 50-ml tube containing HBSS and pen/strep (penicillin/streptomycin, 100 U/ml/100 µg/ml; Thermo Scientific). Each brain was then removed under stereoscope and placed in a clean Petri dish, the meninges carefully detached, and the entire forebrain placed in a 1.5-ml tube following removal of the olfactory bulbs. The tissues were incubated with the non-enzymatic dissociation buffer (Sigma-Aldrich) for 15 min at 37 °C. The cells were then centrifuged 400 × g and the cellular pellets resuspended.

The cells were seeded in suspension in NSC medium (DMEM/F12 GlutaMAX 1 x; 8 mmol/L HEPES; 100 U/ml/100 μg/ml Penicillin/Streptomycin; 1 × B27; 1 × N2; 20 ng/mL bFGF; 20 ng/mL EGF; Thermo Scientific) at a density of ten cells/µl until neurospheres of 100–200 µm were formed. These spheres were then mechanically dissociated and seeded again at the same density in the OPC medium (DMEM/F12 GlutaMAX 1 × ; 8 mmol/L HEPES; 100 U/ml/100 μg/ml Penicillin/Streptomycin; 1 × B27; 1 × N2; 20 ng/mL bFGF; 20 ng/mL PDGF; Thermo Scientific). When the spheres reached a diameter of 100–200 µm once more, they were dissociated in single cell suspension and plated in 96-well plates. The cells were then plated at a density of 3000 cells/cm^2^ on poly-d,l-ornithine (50 µg/ml)/laminin (5 µg/ml; Sigma-Aldrich) coating, in OPC medium.

To induce oligodendrocyte differentiation and maturation, the OPC medium was replaced with the oligodendrocyte differentiation medium (DMEM/F12 GlutaMAX 1 × ; 8 mmol/L HEPES; 100 U/ml/100 μg/ml Penicillin/Streptomycin; 1 × B27; 1 × N2; 50 nM T3; 10 ng/ml CNTF; 1 × *N*-acetyl-l-cysteine—NAC; Thermo Fisher Scientific) 3 DIVs after adhesion seeding.

### Oxygen and glucose deprivation (OGD)

Oxygen deprivation was achieved using an airtight hypoxia chamber (Billups-Rothenberg Inc., Del Mar, CA) saturated with 95% N_2_/5% CO_2_ [19], while glucose deprivation was achieved using a glucose-free medium (Gibco). Primary cortical neural cultures were exposed to OGD at 7 DIV (day in vitro), while OPCs were exposed to differentiation induction by T3 24 h earlier. Oxygen was removed by flushing the hypoxia chamber with N_2_/CO_2_ mixture for 6–8 min at 25 lt/min, and this process repeated after half of the incubation time. OGD was maintained for three hours, after which the plates were re-oxygenated in a cell incubator in the pre-treatment culture medium (pH 7.4).

### Immunocytochemistry and high content screening analysis

Indirect immunofluorescence was used to identify neurons (beta-III-tubulin), OPCs (NG2), and mature oligodendrocytes (MBP). The following primary antibodies were used: mouse anti-*β*-III-tubulin (R&D system, Trento, IT) 1:3000; rabbit anti-GFAP (Dako) 1:1000; rabbit anti-NG2 (chondroitin sulfate proteoglycan, neural/glial antigen 2, Millipore, Merck S.p.a., Milan, IT) 1:350; rabbit anti-MBP (Myelin Basic Protein, Dako) 1:500. As secondary antibodies, donkey Alexa 488-conjugated anti-mouse IgG and donkey Alexa 568-conjugated anti-rabbit IgG, 1:500 (Invitrogen, Carlsbad, CA, USA) were used. Cells were also incubated with the nuclear dye Hoechst 33,258 (1 μg/mL in PBS, 0.3% Triton-X 100) to detect nuclei.

All analyses were performed using the Cell Insight™ CX5 High Content Screening platform (HCS; Thermo Fisher Scientific) to quantify condensed nuclei as an indicator of cell death. For OPC cultures, the percentages of NG2- and MBP-positive cells were measured to quantify the culture differentiation.

Analysis was performed as has previously been published in detail [18]. In brief, using the Compartmental Analysis BioApplication software, the algorithm identifies each cell as an object based on nuclear staining. By analyzing the size of each nucleus and the intensity of the Hoechst staining, it calculates the percentage of high intensity/small-sized condensed nuclei against the total number of nuclei in the well. The software is also able to detect the presence of the marker-specific staining in the cell body, calculating the percentage of immunoreactive cells.

The HCS system permits an analysis of the entire culture, avoiding the bias in choosing random fields. A number of 10,000 to 50,000 cells/well was analyzed in each replicate.

### Statistical analysis

The data are expressed as mean ± SD or mean ± SEM. For in vitro studies on neural cells, three replicates have been included for each group and experiment. Student’s t-test was used to compare the quantity of growth factors secreted by the adMSCs derived from the same subject, but cultured at different pH levels. One-way ANOVA, followed by the Tukey’s post-test, was used to analyze statistical differences in the cell-based functional effect of exposure to the adMSC-conditioned medium. GraphPad Prism Software (v.9) was used to analyze the data, and results were considered significant when the probability of their occurrence as a result of chance alone was less than 5% (*p* < 0.05).

## Results

### Donors

The cells used in the study were derived from the intact adipose tissue of ten subjects undergoing abdominal plastic surgery or reduction mammoplasty. The main clinical features of the donors are shown in Table 1. Nine females and one male were included, ranging in age from 22 to 61 (mean ± SD: 39 ± 11). BMI was in the normal weight range in two individuals, in the overweight range in four individuals, and in the obese range in four individuals. The donors’ medical history showed no other significant clinical data.

### adMSC isolation, characterization, and in vitro expansion

adMSCs were successfully isolated from mammoplasty or abdominoplasty-derived tissue, showing the typical fibroblast-like morphology 4 days after seeding. The time required to reach primary confluence was similar for all samples apart from one (sample code 6113), in which cells showed a longer doubling time (Additional file 1: Fig. S1A, B). Human adMSC immunophenotypic characterization was assessed by flow cytometry on adMSCs harvested at passage 3, and adMSCs were characterized by the positive expression of surface antigens CD34, CD44, CD90, CD73, CD105 and CD166, and the absence of CD14, CD45, CD106 and HLA-DR (Table 2). Despite the cultures having been obtained from different individuals, the immunophenotype of the adMSC cells was fairly homogenous, except for CD34 expression levels. Indeed, it has been previously demonstrated that depending on donor characteristics and cell passage number, the percentage of CD34-positive adMSCs varied [20–25].

**Table 2:** Immunophenotypic characterization of human adMSCs

Subject ID	CD44	CD90	CD105	CD166	CD73	CD14	CD34	CD45	CD106	HLA-DR
5898	95.5	91.3	97.2	94.0	98.6	0.5	33.6	0	1.7	0
5903	99.1	81.4	99.3	83.5	91.9	0	11.3	0	0.5	2.2
5919	98.8	98.1	99.3	99.3	93.9	0	6.6	1.2	7.1	3.0
5959	99.6	96.6	99.1	99.2	97.1	0	17.7	0.1	0.6	1.2
5982	99.0	98.0	99.2	99.1	93.9	0	8.1	0	0.9	0.3
6006	99.2	95.0	99.0	98.9	88.3	0.2	3.2	0	0.2	0
6008	97.3	95.9	99.4	99.6	94.6	0	3.5	1.7	1.2	6.7
6038	99.9	94.7	95.5	97.0	94.7	0	0.2	0.2	0	0.1
6111	98.4	96.9	95.9	93.3	86.7	0	0.1	0.5	0	0
6113	98.0	98.8	97.5	97.8	91.4	0	1.4	0.8	0.2	0

Data are expressed as % of positive cells

Additionally, we evaluated the ability of adMSCs to differentiate in osteogenic and adipogenic lineage. After 21 days of culture with specific inductors, the osteogenic differentiation was revealed by the production of a mineralized extracellular matrix positively stained with Alizarin Red (Additional file 1: Fig. S2A). Moreover, cells treated with adipogenic medium contained single lipid droplets stained by Oil Red O (Additional file 1: Fig. S2B).

Conditioned media (5 ml) was harvested from semi-confluent cells (T25) at passage 3, after 72 h of conditioning (Additional file 1: Fig. S3A). The cell density was measured when the cell culture supernatant was collected, and ranged from 7.44 to 15.8 × 10^3^ cells/cm^2^ (Additional file 1: Fig. S3B).

The absence of mycoplasma contamination in the conditioned media harvested from the adMSCs was also verified (Additional files 1, 2: Fig. S3C). No bacteria contamination was detected by plate.

### Growth factor concentration in the conditioned media

**Fig. 1:**
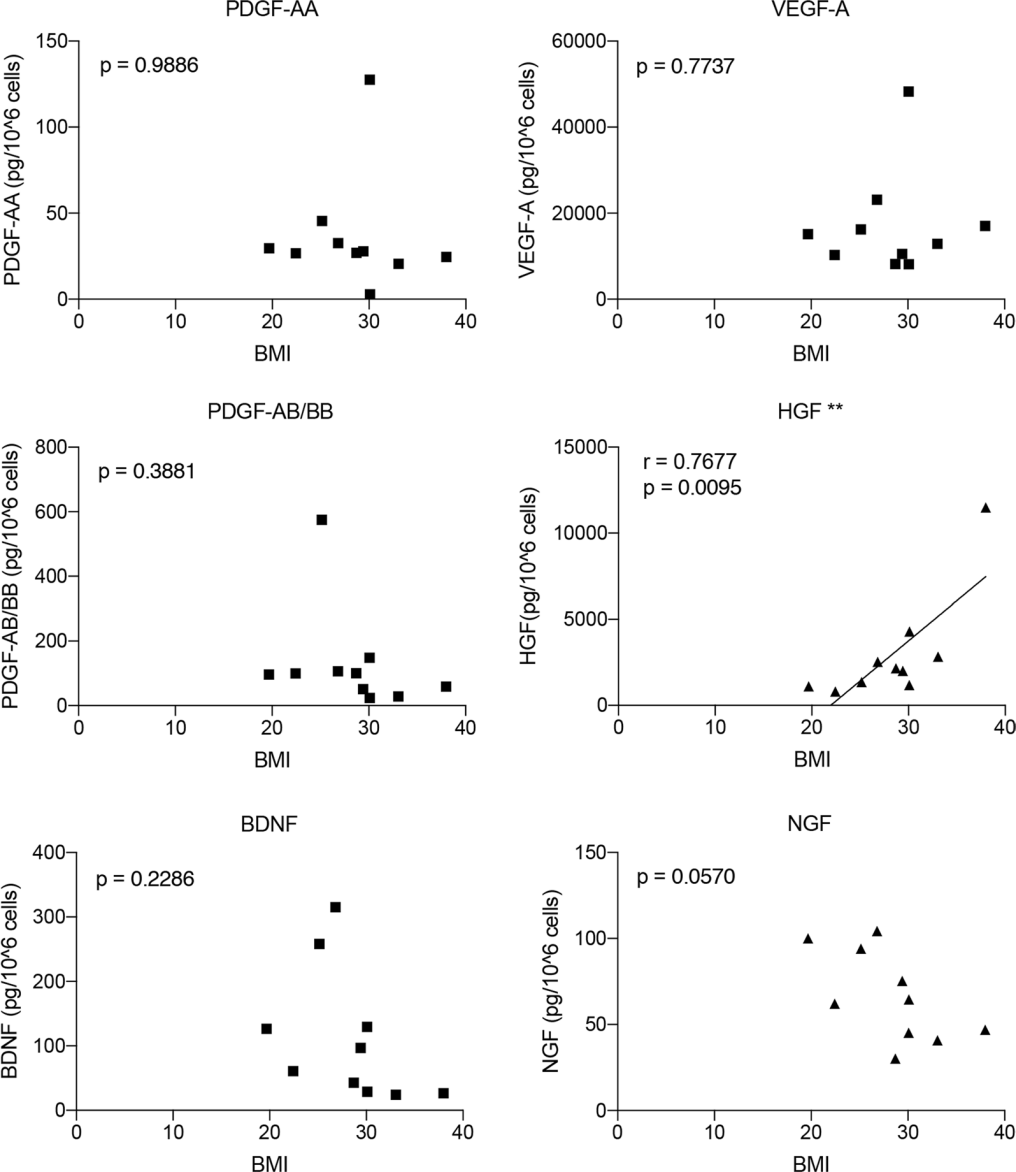
Correlation between the BMI and dosed growth factors. Graphs show the correlation analysis between the amount (pg/10^6^ cells) of dosed VEGF-A, BDNF, HGF PDGF-AA, PDGF-AB/BB and NGF. Only HGF shows a significant positive correlation. Statistical analysis. Dots represent the average values of the replicates, while the lines represent the simple linear regression (only showed where correlation is significative). Pearson’s R test, *r* and *p* values are included in each graph

Growth factor concentration was measured over a 72-h culture period, and individual results are shown in Table 3 as pg/ml (mean ± SEM of two different aliquots/individual). The adMSC values in the conditioned media from the commercial cell lines purchased from ATCC (COMM1) and LONZA (COMM2) are also included.adMSCs secreted significant amounts of PDGF-AA, VEGF-A, PDGF-AABB, HGF, BDNF and NGF, whereas FGF-21, GDNF, IGF-I, IGF-II, EGF and FGF-2 were undetectable in all conditioned media tested. The level of PDGF-AA ranged from 0.17 to 9.19 pg/ml; VEGF-A from 447.57 to 3473.04 pg/ml; PDGF-AABB from 1.48 to 21.38 pg/ml; HGF from 50.67 to 551.85 pg/ml; BDNF from 1.27 to 63.16 pg/ml, and NGF from 2.15 to 10.65 pg/ml, thus showing an individual variability ranging from 4.95 times (ratio maximum/minimum value, NGF) to 52.70 times (ratio maximum/minimum value, PDGF-AA). At medium collection, the cell count showed 288.250 cells on average, with a percentage variability of less than 25%. In Additional file 3: Table S1, the growth factor quantification results are normalized on the number of cells at the time of harvest. In order to establish if the observed in growth factors levels variability it could depend on the pathological conditions of the subjects, such as obesity, we performed a correlation analysis between BMI and analytes concentration (pg/10^6^cells). HGF shows correlation with BMI (Pearson correlation, *p* = 0.0095, Fig. 1).

**Table 3:** Levels of detectable growth factors in media conditioned with adMSCs for 72 h

Subject ID	BDNF	PDGF-AA	PDGF-AB/BB	NGF	VEGF-A	HGF
5898	1.27 ± 0.19	1.18 ± 0.29	2.83 ± 0.00	2.25 ± 0.07	817.82 ± 24.55	551.85 ± 6.43
5903	3.35 ± 0.18	2.10 ± 0.18	7.80 ± 1.03	2.35 ± 0.21	637.63 ± 54.98	167.91 ± 8.51
5919	1.79 ± 0.56	0.17 ± 0.19	1.48 ± 0.00	4.00 ± 0.28	505.21 ± 69.73	266.33 ± 30.01
5959	4.11 ± 0.18	1.18 ± 0.10	2.16 ± 0.96	3.20 ± 0.14	447.57 ± 18.16	85.03 ± 0.40
5982	1.27 ± 0.19	1.08 ± 0.05	1.48 ± 0.00	2.15 ± 0.07	676.29 ± 19.84	148.69 ± 5.46
6006	14.65 ± 0.17	1.52 ± 0.09	4.93 ± 1.00	4.85 ± 0.35	1074.80 ± 124.49	117.57 ± 6.34
6008	8.34 ± 0.17	1.95 ± 0.11	6.36 ± 1.02	6.60 ± 2.12	999.22 ± 28.69	73.24 ± 1.64
6038	9.61 ± 0.56	1.69 ± 0.05	21.38 ± 0.00	3.50 ± 0.00	603.57 ± 14.90	50.67 ± 0.00
6111	9.32 ± 0.48	9.19 ± 0.03	10.67 ± 3.93	3.25 ± 0.35	3474.04 ± 77.83	85.20 ± 1.44
6113	4.79 ± 0.16	2.10 ± 0.04	7.89 ± 0.00	4.90 ± 0.71	812.21 ± 49.76	63.45 ± 0.00
COMM1	43.72 ± 1.37	2.60 ± 0.08	11.09 ± 0.66	10.65 ± 1.34	1968.36 ± 98.67	76.98 ± 4.38
COMM2	63.16 ± 5.49	6.85 ± 0.54	10.63 ± 1.31	5.40 ± 0.85	2616.51 ± 85.36	92.32 ± 2.86

Data are expressed as pg/ml (mean ± SEM obtained from two different aliquots for each individual or commercial cells)

FGF-21, GDNF, IGF-I, IGF-II, FGF2 and EGF were undetectable in all tested media

Inflammation is a common event in most neurodegenerative conditions, and an anti-inflammatory role has been attributed to the secretome of MSCs. As tissue and local extracellular acidification are associated to inflammation and ischemia (PMID: 25,461,841; PMID: 23,530,046), we decided to evaluate whether acidic conditions affect growth factor production, and, consequently, their potential therapeutic activity. Cells taken from three subjects with BMI values in the normal (6008), overweight (6006) and obese (5982) ranges, and different growth factor concentrations measured in adMSC-derived conditioned media, were also cultured in acidic pH (pH = 6.8) and growth factors measured. We initially investigated whether different pH values affect the growth factor assay, comparing standard curves at pH 7.4 and pH 6.8 (Additional file 1: Fig. S4). All interpolating curves show *r*^2^ > 0.98, indicating no pH effect on assay. Some single standard concentrations, however, show differences based on pH (indicated with asterisks in the graphs). Each sample has therefore been interpolated on the standard curve of conditioned medium at the same pH.

**Fig. 2:**
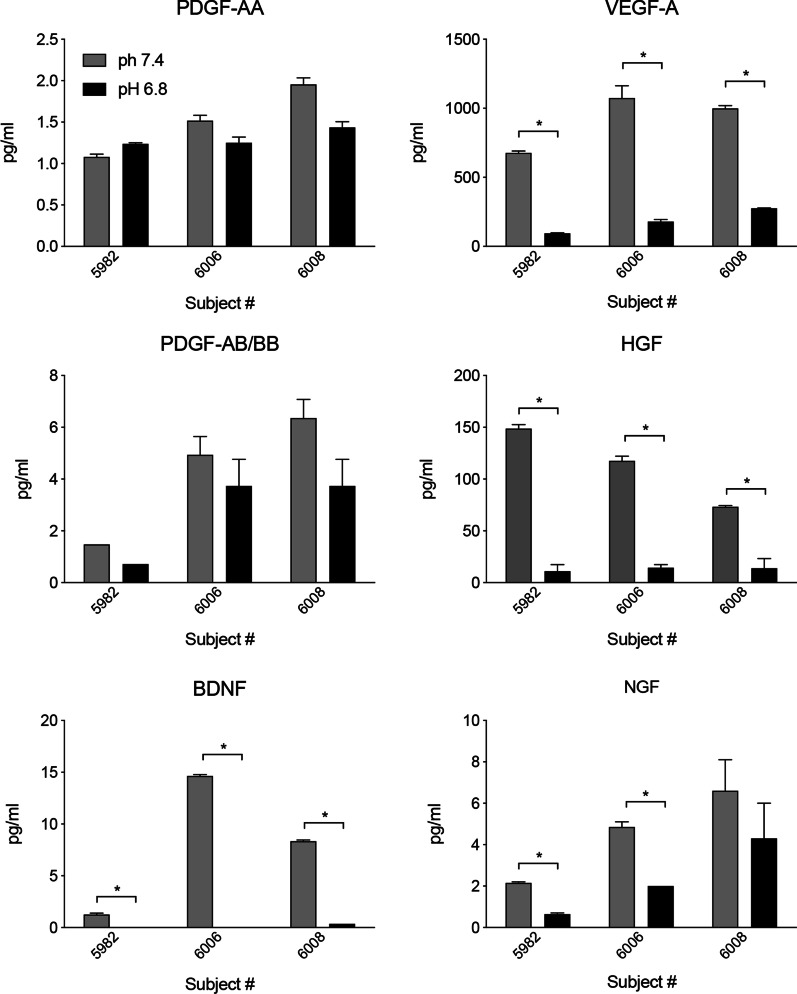
Effects of low and high pH on growth factor production. Bars represent growth factor levels measured in media collected from the same subject, cultured at different pH. Data are expressed as mean ± SEM. Statistical analysis: *t*-test. Asterisks represent the differences between the indicated groups (*p < 0.05)

AdMSCs also secreted significant amounts of PDGF-AA, VEGF-A, PDGF-AABB, HGF, BDNF, NGF in acidic conditions, whereas FGF-21, GDNF, IGF-I, IGF-II, EGF and FGF-2 were undetectable in all tested media. As shown in Fig. 2, the production of VEGF-A, HGF, NGF and BDNF was reduced in acidic conditions, ranging from 54.46% (NGF) to 98.58% (BDNF). Two of the three acidic media tested showed undetectable levels of BDNF.

### In vitro neuroprotection by individual conditioned media

**Fig. 3:**
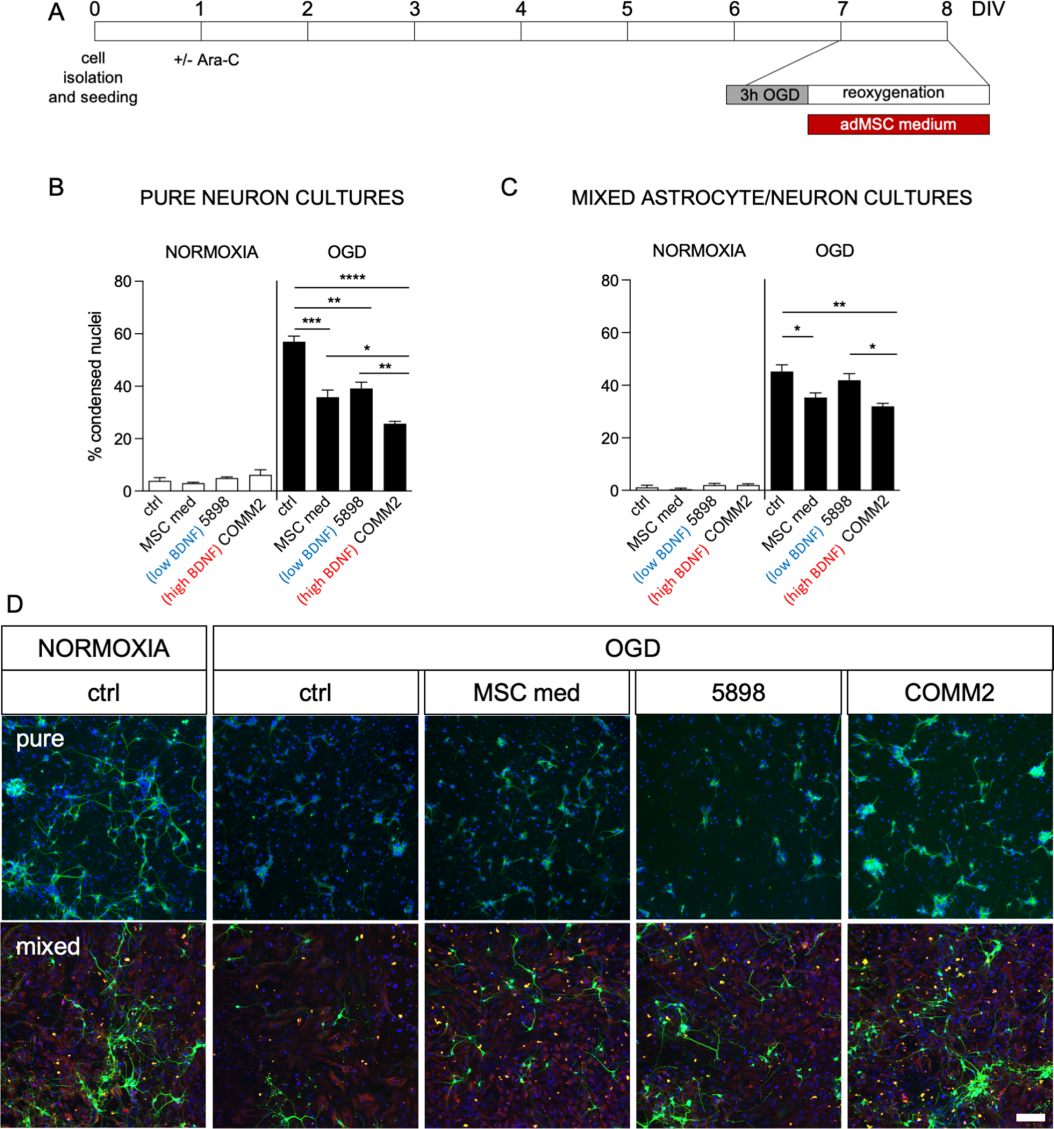
Effect of MSC-conditioned medium containing high and low levels of BDNF on cortical neuron cultures exposed to OGD. **A** The diagram shows the protocol used. Cultures were exposed at 7 DIVs to 3 h of OGD and 24 h of reoxygenation. Cells were cultured in standard medium (ctrl), mesenchymal stem cell medium (MSC med), or conditioned medium obtained from donor 5898 (low BDNF) or COMM2 (high BDNF) samples during the reoxygenation phase for 24 h. At 8 DIVs cells were processed for immunocytochemistry and cell-based HCS analysis. **B**, **C** Graphs show the percentage of condensed nuclei measured in pure neuronal (**B**) or mixed astrocyte/neuron cultures (**C**) exposed to normoxia control conditions or OGD. **D** Representative images of pure neuron and mixed astrocyte/neuron cultures exposed to normoxia under control conditions, or to OGD under control conditions, with the culture medium of mesenchymal stem cells (MSC med), or with the conditioned media. The following stainings were used: beta-III-tubulin (green; neurons), GFAP (red; astrocytes) Hoechst (blue; nuclei). Images were modified increasing brightness for visualization purposes, but High Content Screening software process the raw images independently from post-acquisition modifications. The same brightness/contrast manipulation was applied to the whole image set. Scale bar: 200 µm. Statistical analysis. Columns represent the mean + SEM. One-way ANOVA followed by Tukey’s post-test. Asterisks represent the differences between the indicated groups (*p < 0.05; **p < 0.01; ***p < 0.001; ****p < 0.0001)

To investigate the neuroprotective properties of the MSC secretome, we first selected two different conditioned media, corresponding to low (1.27 pg/ml, subject 5898) and high (63.16 pg/ml, COMM2 cells) BDNF levels, and tested their efficacy at protecting neurons from OGD insult (Fig. 3A), given that this molecule is a well-known trophic factor for cortical neurons [26]. We used both pure neurons and mixed astrocyte/neuron cultures, with standard neuronal medium (ctrl) and an MSC medium (MSC med) as the control. Three replicates per group were added in the experiment. The readout was obtained using HCS, detecting the nuclei of neurons by the presence of the surrounding b-III-tubulin staining, and evaluating cell death as the percentage of condensed nuclei. Results are shown as the mean + SEM between three wells, and approximately 50,000 cells were included in the analysis.

In the pure neuron cultures, none of the tested media and secretomes showed any effects under control normoxia conditions, while a significant reduction in cell death was detected between the groups following OGD exposure (one-way ANOVA, F(3,8) = 36.65, *p* < 0.0001). In particular, while all tested media produced a significant reduction in cell death compared to standard cultures (Tukey’s post-test, MSC med, *p* = 0.0005; 5898, *p* = 0.0017; COMM2, *p* < 0.0001), the secretome containing higher levels of BDNF (COMM2) was more effective at reducing the percentage of condensed nuclei compared to the MSC medium (*p* = 0.0426) and the secretome containing lower levels of BDNF (5898, *p* = 0.0095) (Fig. 3B).

None of the treatments affected cell death under normoxia conditions in mixed astrocyte/neuron cultures either, while a significant variation was detected in OGD (one-way ANOVA, F(3,8) = 8.314; *p* = 0.0077). Compared to standard cultures, the MSC medium and conditioned medium containing high levels of BDNF (COMM2) were both able to reduce the percentage of condensed nuclei (Tukey’s post-test, MSC med, *p* = 0.0412; COMM2, *p* = 0.0089). The conditioned medium containing low levels of BDNF (5898) produced no effect, thus differing significantly from the medium obtained from COMM2 (*p* = 0.0408) (Fig. 3C).

Representative images obtained from the HCS acquisitions are shown in Fig. 3D.

### Effect of individual conditioned media on OPCs

**Fig. 4:**
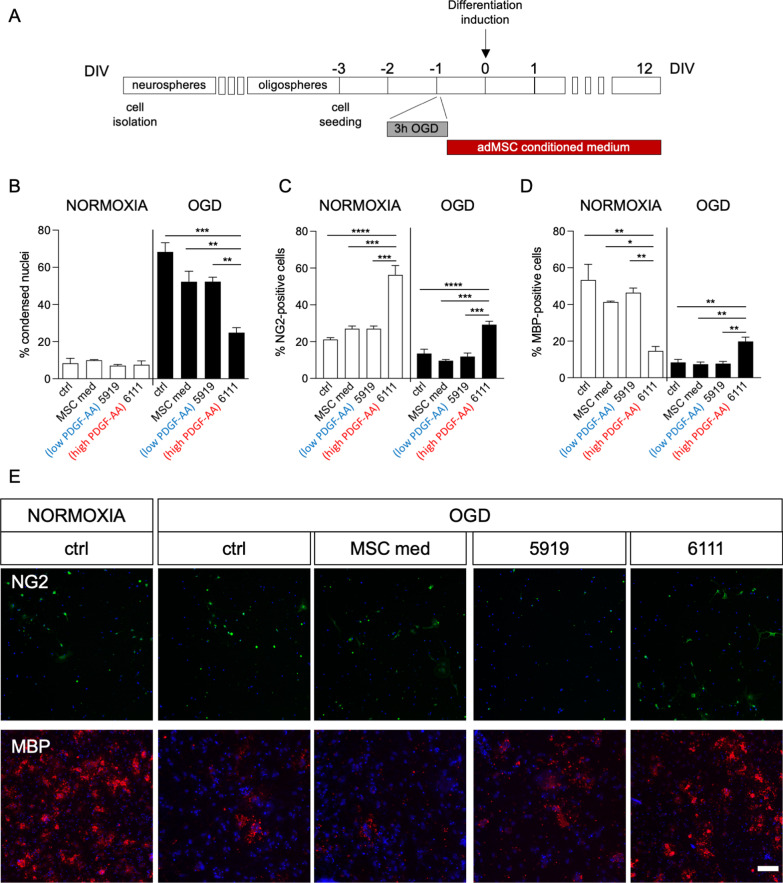
Effect of MSC-conditioned medium containing high and low levels of PDGF-AA on oligodendrocyte precursor cells exposed to OGD. **A** The diagram shows the protocol used. After the suspension phases (neurosphere and oligosphere), NSC-derived OPCs were seeded in adhesion as undifferentiated precursors ( − 3 DIV). After 2 DIVs, cultures were exposed to OGD for 3 h, followed by a further DIV differentiation induced by addition of T3. Cells were cultured in standard medium (ctrl), mesenchymal stem cell medium (MSC med), or conditioned medium obtained from donor samples 5919 (low PDGF-AA) or 6111 (high PDGF-AA), following OGD exposure throughout the differentiation. At 12 DIVs after differentiation induction, cells were processed for immunocytochemistry and cell-based HCS analysis. **B** Graph shows the percentage of condensed nuclei measured in OPC cultures exposed to normoxia control conditions or OGD. Cells were cultured in standard medium (ctrl), mesenchymal stem cell medium (MSC med), or conditioned medium obtained from donor samples 5919 (low PDGF-AA) or 6111 (high PDGF-AA). **C**, **D** Graphs show the percentage of NG2- (**A**) or MBP- (**B**) positive cells in OPC cultures exposed to normoxia control conditions or OGD. Cells were cultured in standard medium (ctrl), mesenchymal stem cell medium (MSC med), or conditioned medium obtained from donor samples 5919 (low PDGF-AA) or 6111 (high PDGF-AA). **E** Representative images of OPC cultures exposed to normoxia under control conditions, or to OGD under control conditions, with the culture medium of mesenchymal stem cells (MSC med), or conditioned media. The following stainings were used: NG2 (green; OPC), MBP (red; mature oligodendrocytes) Hoechst (blue; nuclei). Images were modified increasing brightness for visualization purposes, but High Content Screening software process the raw images independently from post-acquisition modifications. The same brightness/contrast manipulation was applied to the whole image set. Scale bar: 200 µm. Statistical analysis. Columns represent the mean + SEM. One-way ANOVA followed by Tukey’s post-test. Asterisks represent the differences between the indicated groups (*p < 0.05; **p < 0.01; ***p < 0.001; ****p < 0.0001)

We used the same challenge on NSC-derived fetal OPC cultures, choosing two different MSC-conditioned media for the treatment, one with low and the other with high levels of PDGF-AA (0.17 pg/ml, subject 5919; 9.19 pg/ml, subject 6111, respectively), a well-known trophic and differentiation factor for OPCs [27] (Fig. 4A). We used two different parameters to evaluate the protective and differentiating effects of the conditioned media: i) percentage of condensed nuclei, as a quantification of cell death, and ii) percentage of cells positive for specific markers to identify OPCs (NG2) and mature oligodendrocytes (MBP). Three replicates per group were added in the experiment.

Cell death was not affected under normoxia conditions, while a significant reduction in the percentage of condensed nuclei was detected under OGD (one-way ANOVA, F(3,8) = 18.75, *p* = 0.0006). In particular, the medium conditioned by sample 6111 (high PDGF-AA concentration) resulted in a strong reduction in cell death, significantly different to all other groups (Tukey’s post-test, ctrl, *p* = 0.0004; MSC med, *p* = 0.0072; 5919, *p* = 0.0070) (Fig. 4B).

The percentage of undifferentiated precursors was modified by the treatment both in normoxia (one-way ANOVA, F(3,8) = 32.23, *p* < 0.0001) and OGD (one-way ANOVA, F(3;8) = 23.88, *p* = 0.0002), with an increase resulting from exposure to medium 6111 (Tukey’s post-test; normoxia, ctrl, *p* < 0.0001; MSC med, *p* = 0.0003; 5919, *p* = 0.0003; OGD, ctrl, *p* = 0.0014; MSC med, *p* = 0.0003; 5919, *p* = 0.0007) (Fig. 4C).

The medium conditioned by sample 6111, while causing a reduction of mature oligodendrocytes under normoxia conditions compared to all other groups (one-way ANOVA, F(3,8) = 13.37, *p* = 0.0018; Tukey’s post-test, ctrl, *p* = 0.0016; MSC med, *p* = 0.0149; 5919, *p* = 0.0056), exerted a protective effect after OGD, showing an increase in the percentage of MBP-positive cells (one-way ANOVA, F(3,8) = 13.25, *p* = 0.0018; Tukey’s post-test, ctrl, *p* = 0.0052; MSC med, *p* = 0.0031; 5919, *p* = 0.0037) (Fig. 3D).

Representative images obtained from the HCS acquisitions are shown in Fig. 4E.

### Correlation analysis

To establish whether the observed donor-related differences in growth and neurotrophic factor concentration correlate with neuron protection and/or OPC protection and maturation, in another set of experiments we performed the same in vitro tests as in Figs. 3 and 4, choosing the conditioned media with the highest and lowest BDNF levels for pure neuron cultures (5898, 6006, 5919, 5982, 6008, COMM1, COMM2), and the highest and lowest PDGF-AA levels for OPCs (5919, 5982, 5898, 5903, 6111, COMM2).

**Fig. 5:**
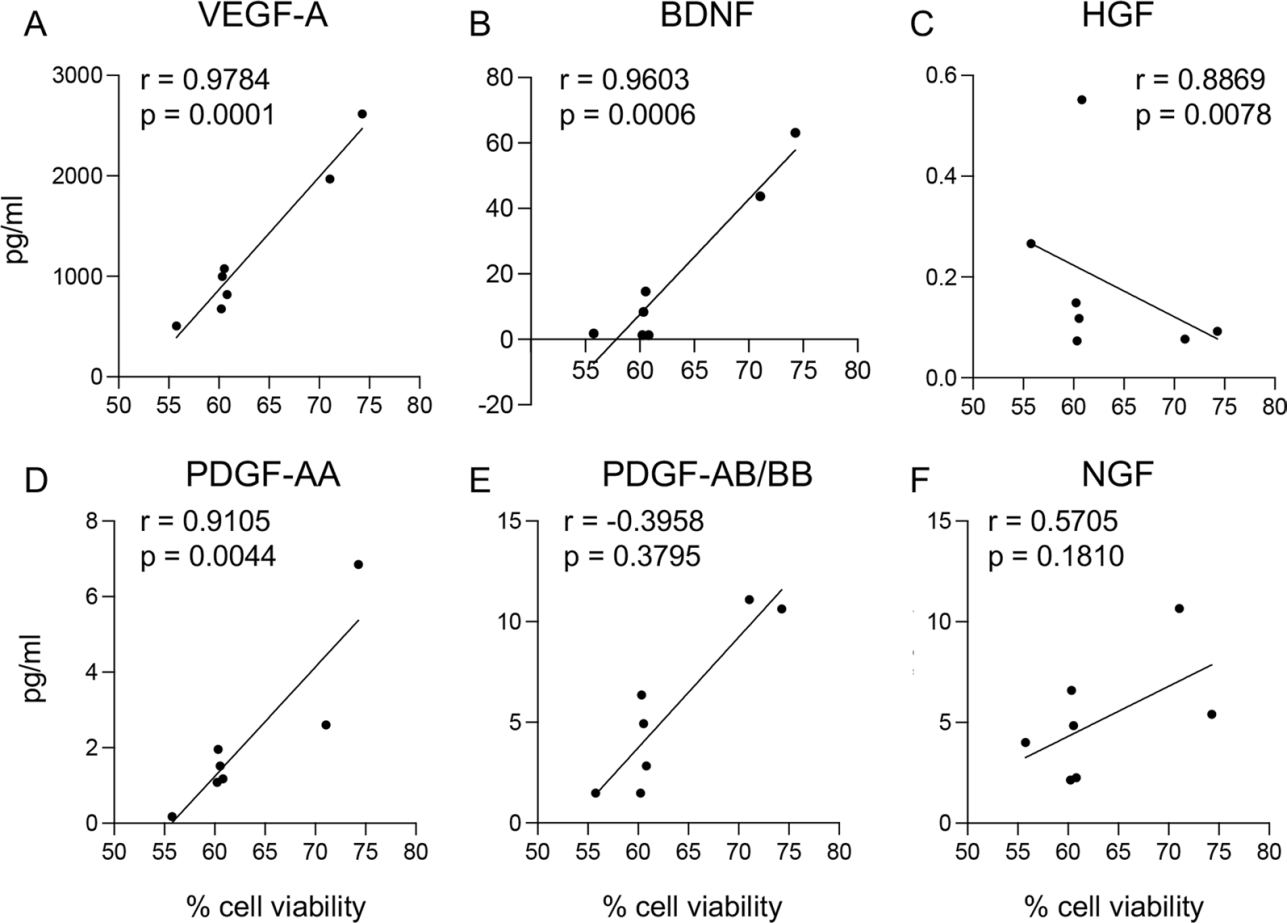
Correlation between the dosed growth factors and neuroprotective effect. **A**, **F** Graphs show the correlation analysis between the biological effect of the conditioned medium, as neuroprotection against OGD in pure neuronal cultures, and the amount of dosed VEGF-A (**A**), BDNF (**B**), HGF (**C**), PDGF-AA (**D**), PDGF-AB/BB (**E**) and NGF (**F**). Statistical analysis. Dots represent the average values of the replicates, correlating the two parameters, while the lines represent the simple linear regression. Pearson’s R test, *r* and *p* values are included in each graph

Neuron viability following OGD insult showed a positive correlation with VEGF-A, BDNF and PDGF-AA detected concentrations, and an inverse correlation with HGF, while no correlation was found for NGF and PDGF-AB/BB levels (Fig. 5A–F).

**Fig. 6:**
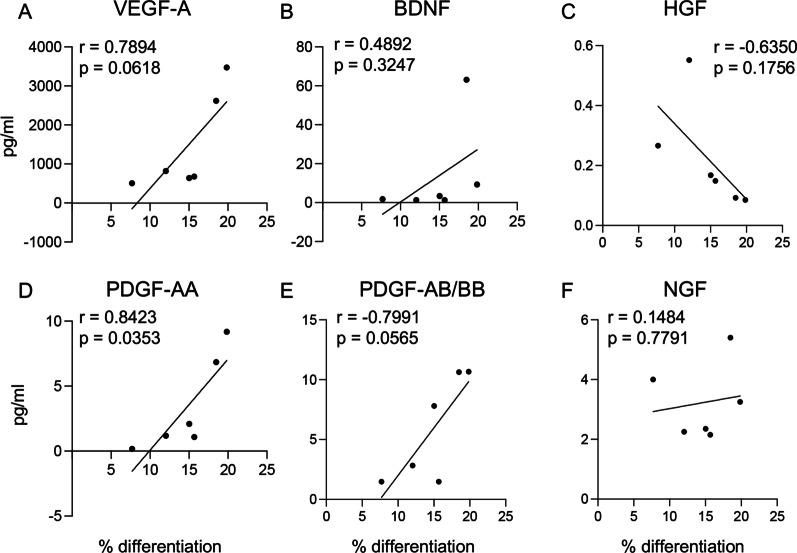
Correlation between the measured growth factors and protective effect of OPCs. **A**, **F** Graphs show the correlation analysis between the biological effect of the conditioned medium, as OPC differentiation protection against OGD, and the amount of VEGF-A (**A**), BDNF (**B**), HGF (**C**), PDGF-AA (**D**), PDGF-AB/BB (**E**) and NGF (**F**) measured. Statistical analysis. Dots represent the average values of the replicates, correlating the two parameters, while the lines represent the simple linear regression. Pearson’s R test, *r* and *p* values are included in each graph

For the readout of the OPC differentiation after OGD, a positive correlation emerged for PDGF-AA levels only, while no correlations were found with VEGF-A, BDNF, HGF, PDGF-AB/BB or NGF concentrations (Fig. 6A–F).

## Discussion

The available preclinical evidence from different experimental models of nervous system lesions and degenerative diseases supports an immunomodulatory and trophic action related to the secretome of MSCs, capable of changing disease outcome at functional, anatomical and/or molecular level (4,5). Adipose tissue-derived MSCs produce a broad array of cytokines, chemokines (both pro-inflammatory and anti-inflammatory), adipokines, antioxidative molecules, pro-angiogenic factors, anti-apoptotic factors, growth factors (VEGF, HGF, FGF-21, IGF-1, EGF and FGF-2), neurotrophic factors (BDNF, NGF) and interleukins (IL-1Ra, IL-6, IL-7, IL-8, IL-11) [28], but also contain different types of extracellular vesicles with neuroprotective and neurotrophic effects [29] and miRNA [30], prompting an interest in MSCs and MSC-derived acellular products (secretome, different types of extracellular vesicles, conditioned culture medium) as candidate drugs for nervous system lesions and diseases [31]. Translation of the data to clinical practice, however, is currently hampered by a lack of consistency and coherence, while the recent report of the phase 2 MEsenchymal StEm cells for Multiple Sclerosis (MESEMS) clinical trial, devised to evaluate the safety, tolerability and activity of autologous MSCs derived from bone marrow and infused intravenously in patients with active multiple sclerosis, does not support the use of bone marrow-derived-MSCs to treat active multiple sclerosis [32], further increasing the complexity of the puzzle.

Given that the donor-dependent variations in MSC properties is a still a poorly explored aspect of preclinical to clinical translation, in this study we prepared adMSC-conditioned media using cells from ten individuals from a fairly homogenous population and undergoing reductive abdominal and breast surgery. Two commercial cell lines were also included. BMI values were in the normal (two subjects), overweight (4 subjects), obese (3 subjects) and extremely obese ranges (1 subject): although there is evidence that this clinical feature affects MSC characteristics, the available data are somewhat contrasting. While a lower proliferation rate and colony formation ability, such as a lower osteogenic and adipogenic differentiation potential has been described [14], other researchers have observed an increased proliferation rate for MSCs isolated from obese subjects [33], as observed in our own study (subject 5898), while MSCs derived from obese patients were less prone to the anti-inflammatory and immunomodulatory properties compared to derived from non-obese subjects when implanted in an animal model of multiple sclerosis [34].

We initially analyzed growth factor concentration in the medium, focusing on neurotrophic and growth factors of interest for CNS protection and repair. In a similar way to MSCs derived from other tissue sources (bone marrow, umbilical cord, fetal membranes, dental pulp), adipose MSCs produce a pattern of neuro-regulatory molecules [22, 35, 36]. Using a cell density of around 250 × 10^3^/5 ml, we confirmed the secretions of several growth and neurotrophic factors in culture media at pg/ml range concentration, including PDGF-AA, VEGF-A, PDGF-AABB, HGF, BDNF and NGF, whereas FGF-21, GDNF, IGF-I, IGF-II, EGF and FGF-2 were undetectable. Although culture conditions (cell density, passage number) can affect the degree of growth factor production, and although different quantification methods can lead to variability of the results, our data confirm what has already been observed in the literature. The growth factor levels produced by mesenchymal stem cells range, in fact, between 50 and 25,000 pg/10^6^ cells, both from cells derived from adipose tissue and from other tissue [37–40].

Individual variability is fairly consistent for all measurable growth factors, being 52.70 times the ratio maximum (donor 6111)/minimum (donor 5919) value for PDGF-AA, for example, a key molecule for remyelination [27, 41]. In contrast, NGF concentrations vary by a maximum of four times between donors (from 2.5 to 10 pg/ml). NGF regulates several aspects of MSC biology such as survival, growth, differentiation and angiogenesis via paracrine mechanisms involving the membrane receptors TRKA and p75 [42]. Notably, NGF production is much higher in bone marrow-derived MSCs [35], and differences in NGF-mediated mechanisms may reflect the different developmental origins of the MSCs populating the tissue source [42]. In cultures derived from commercial cells, we observed that BDNF secretion is 10 times the mean value observed in the other cells. We don't have an explanation for this, and all available information related to the donor and cell characterization provided by the commercial supplier has been reported. A missing information is for example the passage number, that can modify growth factor secretion, as discussed in other parts of the paper. This result also highlights the need of a full knowledge of the cells, in terms of donor, culture and storage condition, not always available from commercial sources.

Finally, since we included an heterogenous population in terms of BMI, we attempted to correlated growth factors secretion with BMI, and one analyte (HGF) of the six analyzed shows positive correlation with BMI. HGF is considered an important component of the pathophysiology of insulin resistance, regulating the metabolic flux of glucose in different insulin-sensitive cell types and playing a role in *β*-cell homeostasis [43]. This result further confirms that BMI must be considered in individual variability of MSCs paracrine properties.

We then explored the neuroprotective and remyelination potential of adMSC-conditioned media, using OGD as challenge. OGD is an experimental in vitro condition used to mimic hypoxia-ischemic insults, widely used in our lab and supported by cell-based High Content Analysis [44, 45]. Testing cells with patient-derived biological fluids has already been used as an approach to predict clinical outcomes [46, 47].

In all these experiments, we also included the MSC medium as control. Control conditions are a vital aspect of this type of research, yet they are not always clear in published results describing the neuroprotective role of MSC-conditioned medium, even when using transwell systems. One key aspect regards the composition of MSC medium, which always contains serum (15–20%), while a serum-free defined mix is usually preferred for neural cultures (both neurons and OPCs). Serum contains an undefined mixture of soluble molecules, including growth factors, which introduce a high degree of variability: adding serum to the standard neuronal medium is therefore not sufficient to create a suitable control, and an MSC medium with exactly the same composition prepared in parallel with the conditioned medium should be used instead [48, 49].

Some studies also use cell lines, which poorly mimic in vivo conditions and responses to noxious stimuli [50, 51].

None of the treatments affected neuron viability in normoxia, while substantial protection by the MSC medium itself was observed in the pure neuronal cultures, an effect which was further increased by the MSC-conditioned medium (COMM2). In mixed cultures, in which the astrocytes already exert neuroprotection, reducing cell death by approximately 15%, the MSC medium offered lower protection, and no substantial differences were observed between the MSC medium and the MSC-conditioned medium.

In OPC cultures, the MSC medium alone was ineffective in both normoxia and OGD, while the secretome from donor 6111, with its high concentration of PDGF, was highly effective at protecting the OPCs and pushing OPC differentiation toward mature OLs under OGD conditions. It is not surprisingly, in fact, that in normoxia condition the high concentration of the PDGF contrasts the T3-mediated differentiation induction, while protecting the OPCs during the noxious stimulus. To the best of our knowledge, this is the first study to describe a direct effect of the human adipose MSC secretome on OPC protection and differentiation and is consistent with a report describing how pro-oligodendroglial factors derived from human fetal MSCs can instruct human-induced pluripotent stem cell-derived NSCs to differentiate into O4 positive oligodendrocytes [52]. These results support in vivo data on the potential of the secretome of MSCs derived from different tissues to improve remyelination in animal models of demyelinating diseases [51, 53, 54]. Notably, the pharmacological concentrations of neuroprotective growth factors in in vitro studies are much higher than in conditioned media, usually used in a range of 10–200 ng/ml (NGF, [55, 56]; BDNF, [57]; PDGF, [58]), thus supporting the view that a complex therapeutic product such as conditioned medium, containing multiple bioactive molecules including proteins but also miRNA, and different formulations (free molecules and extracellular vesicle), are more active than single drugs in improving remyelination.

To explore whether the observed results were related to donor variability, we correlated the growth and neurotrophic factor concentrations in conditioned media composition with the biological effects on neurons and OPCs, demonstrating that the degree of neuroprotection of primary neurons derived from the embryonic telencephalon and exposed to OGD positively correlates with VEGF-A, BDNF and PDGF-AA, and negatively correlates with HGF. BDNF is known to promote neural cell proliferation and survival in the developing brain [59]; VEGF-A has neurotrophic and neuroprotective effects on neuronal and glial cells in culture and in vivo [60], and PDGF suppresses oxidative stress-induced Ca2 + overload in neurons [61]. Even more surprising was the negative correlation we observed between OGD neuroprotection and HGF concentration, HGF having recently been indicated as a key factor in preventing neuronal death and promoting survival through pro-angiogenic, anti-inflammatory, and immune-modulatory mechanisms in vivo [62]. Bone marrow-derived MSCs transfected with HGF also protected neurons against OGD-induced apoptosis [63, 64].

OPC differentiation positively correlates with PDGF-AA and PDGF-AB/BB, as expected based on the pharmacological data on this growth factor and the fact that it is a component of OPC-differentiating culture media [65–67].

Additionally, adMSC were cultured in acidic conditions to mimic the inflammatory status occurring in neurodegenerative conditions, and their secretome was collected. We demonstrated that the concentration of growth factors in acidic conditioned secretome was significantly reduced. These data added further credence to the concept of favoring MSCs-derived acellular product (i.e., secretome) instead of cells therapy, as MSC implanted in a defective site are sensitive to the microenvironment, and their regenerative potential, in terms of secretory activity, can be highly reduced.

This study has several limitations, beginning with the low number of subjects recruited, and the fact that no dose-dependent experiments are presented. Moreover, we investigated only growth factors of potential interest in neuroprotection, excluding for example inflammation-related cytokines and chemokines. Other factors could affect results as the MSC derivation and culture passages. However, we investigated an important aspect in moving MSCs and MSC-derived acellular products from preclinical to clinical translation, and one that is still under-investigated, namely the variability of efficacy data based on individual cell properties. Batch variability is in fact becoming a subject of increased attention with regard to cell therapy manufacturing, but related end-points mainly refer to growth kinetics and immunological phenotype as the main source of heterogeneity between donors [2, 68–71]. We also highlighted the proper controls for these in vitro experiments, such as culture media composition.

## Conclusions

We can conclude that: (1) MSC secretome shows significant individual variability within a fairly homogenous population; (2) remyelination should be a primary target in MSC-derived therapies, being the only true reparative capability of the CNS; (3) a preliminary screening of secretome properties should include growth and neurotrophic factors for both neurons; (4) finally, OPCs and OLs should be recommended in setting best practices for MSC-based therapies, also for the purposes of quality control [1]. Results from this pilot study must be confirmed in a large cohort of subject, to be translated into clinical guidelines.

## Supplementary Information

**Additional file 1.** Representative pictures and growth curve of adMSC (Supplementary Figure 1); representative pictures of in vitro differentiation of adMSCs into osteoblast and adipocytes (Supplementary Figure 2); representative pictures of semi-confluent adMSC samples at passage 3, cell density measurement and mycoplasma contamination PCR test (Supplementary Figure 3); effect of low and high pH on standard curves of detectable growth factors (Supplementary Figure 4).

**Additional file 2.** Original pictures of the PCR gels for the mycoplasma contamination test showed in Supplementary Figure 3C.

**Additional file 3.** Table showing the levels of detectable growth factors in 72-hour conditioned media of adMSC.

## Data Availability

The datasets analyzed during the current study are available from the corresponding author on reasonable request.

## Abbreviations

adMSCs: Adipose tissue-derived mesenchymal stromal cells
ATMPs: Advanced therapy medicinal products
BDNF: Brain derived neurotrophic factor
BMI: Body mass index
COMM: Commercial cell line
DIV: Day in vitro
DMEM: Dulbecco's modified eagle medium
EGF: Epithelial growth factor
FBS: Fetal bovine serum
FGF: Fibroblast growth factor
FITC: Fluorescin isothiocynate
GDNF: Glial derived growth factor
GFAP: Glial fibrillary acidic protein
HADK2MAG-61K: Human adipokine magnetic bead panel 2
HAGP1MAG-12K: Human angiogenesis growth factor magnetic bead panel 1
HCYTA-60K: Human cytokine/chemokine/growth factor panel A
HGF: Hepatocyte growth factor
HIGFMAG-52K: Human IGF-I, II magnetic bead panel
HLPPMAG-57K: Human liver protein magnetic bead panel
HNDG3MAG-36K: Human neurodegenerative disease magnetic bead panel 3
HNDG4MAG-36K: Human neurodegenerative disease magnetic bead panel 4
IGF: Insulin growth factor
MBP: Myelin basic protein
NG2: Neuronal/glia antigen 2
NGF: Nerve growth factor
OGD: Oxygen–glucose deprivation
OPC: Oligodendrocyte precursor cell
PBS: Phosphate-buffered saline
PDGF: Platelet derived growth factor
SVF: Stromal vascular fraction
TRKA: Tropomyosin receptor kinase A
VEGF-A: Vascular endothelial growth factor A
