# Supplementary material for: Intra-individual variability in the neuroprotective and promyelinating properties of conditioned culture medium obtained from human adipose mesenchymal stromal cells
Source: Stem Cell Res Ther. 2023 May 11;14:128. doi: 10.1186/s13287-023-03344-1 (PMC10173531; doi:10.1186/s13287-023-03344-1)
Supplement: Supplementary file 1 — Additional file 1. Representative pictures and growth curve of adMSC (Supplementary Figure 1); representative pictures of in vitro differentiation of adMSCs into osteoblast and adipocytes (Supplementary Figure 2); representative pictures of semi-confluent adMSC samples at passage 3, cell density measurement and mycoplasma contamination PCR test (Supplementary Figure 3); effect of low and high pH on standard curves of detectable growth factors (Supplementary Figure 4). [file 13287_2023_3344_MOESM1_ESM.pdf]

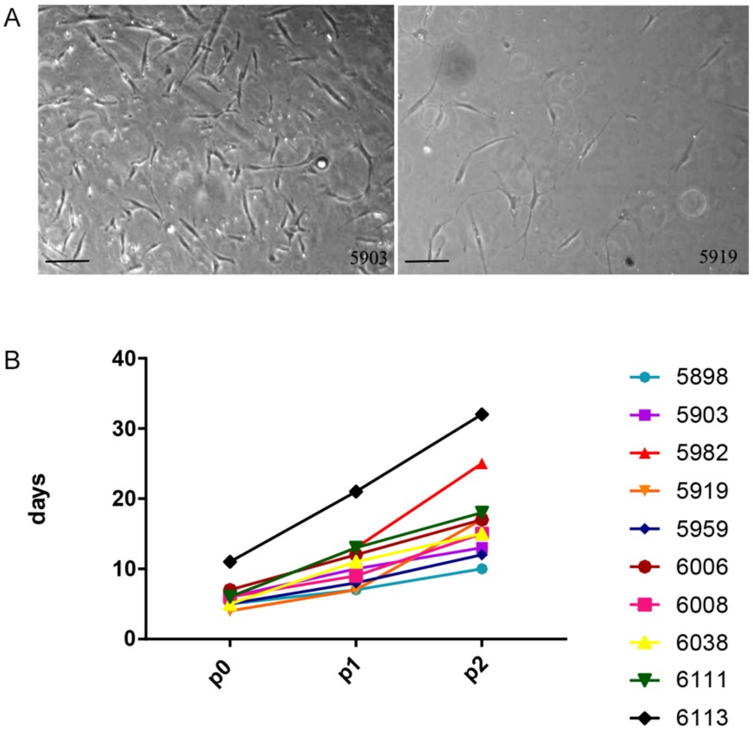


**Supplementary Figure 1.** (A) Representative pictures of 4 days after isolation. AdMSC showed the typical fibroblast-like morphology. Bars: 50 µm. (B) Days to reach cell confluence from passage 0 (p0) to passage 1 and 2 (p1 and 2) of individual samples.
Pictures were acquired with bright field microscope (Eclipse TE 2000-S; Nikon; objectives: (Phl 10×/0.25 WD 7.0) coupled with a digital camera (DS-Fi3, resolution 2880 × 2042, 5.9 Mpixel; Nikon), using the NIS-Elements F software (Nikon).

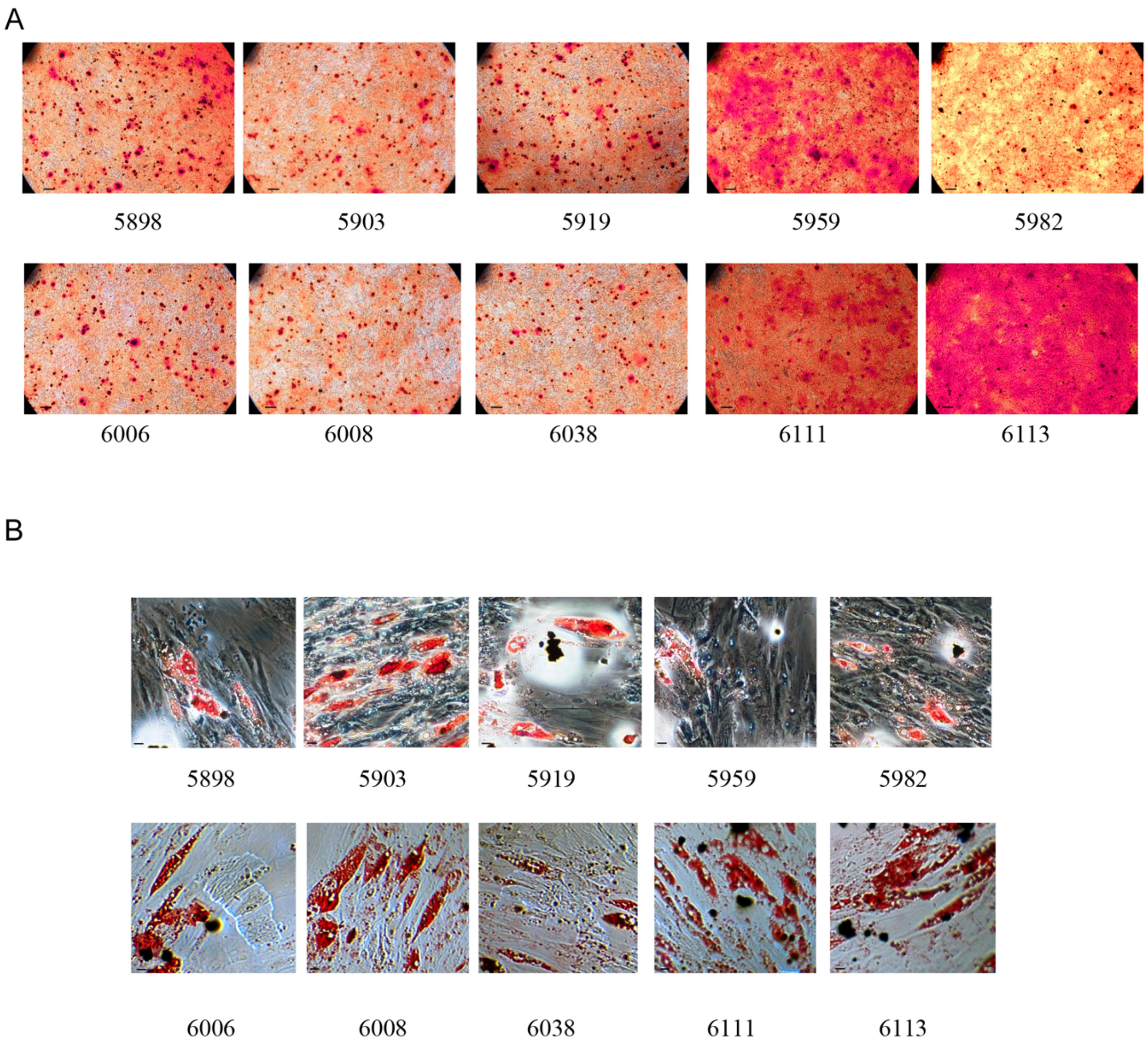


**Supplementary Figure 2.** Representative pictures of in vitro differentiation of adMSCs into osteoblasts (scale bar: 200 µm) (A), and adipocytes (scale bar: 20 µm) (B).
Pictures were acquired with bright field microscope (Eclipse TE 2000-S; Nikon; objectives: (PhlDL 4×/0.13 WD 16.4; ELWD 20×/0.45 WD 7.4) coupled with a digital camera (DS-Fi3, resolution 2880 × 2042, 5.9 Mpixel; Nikon), using the NIS-Elements F software (Nikon).

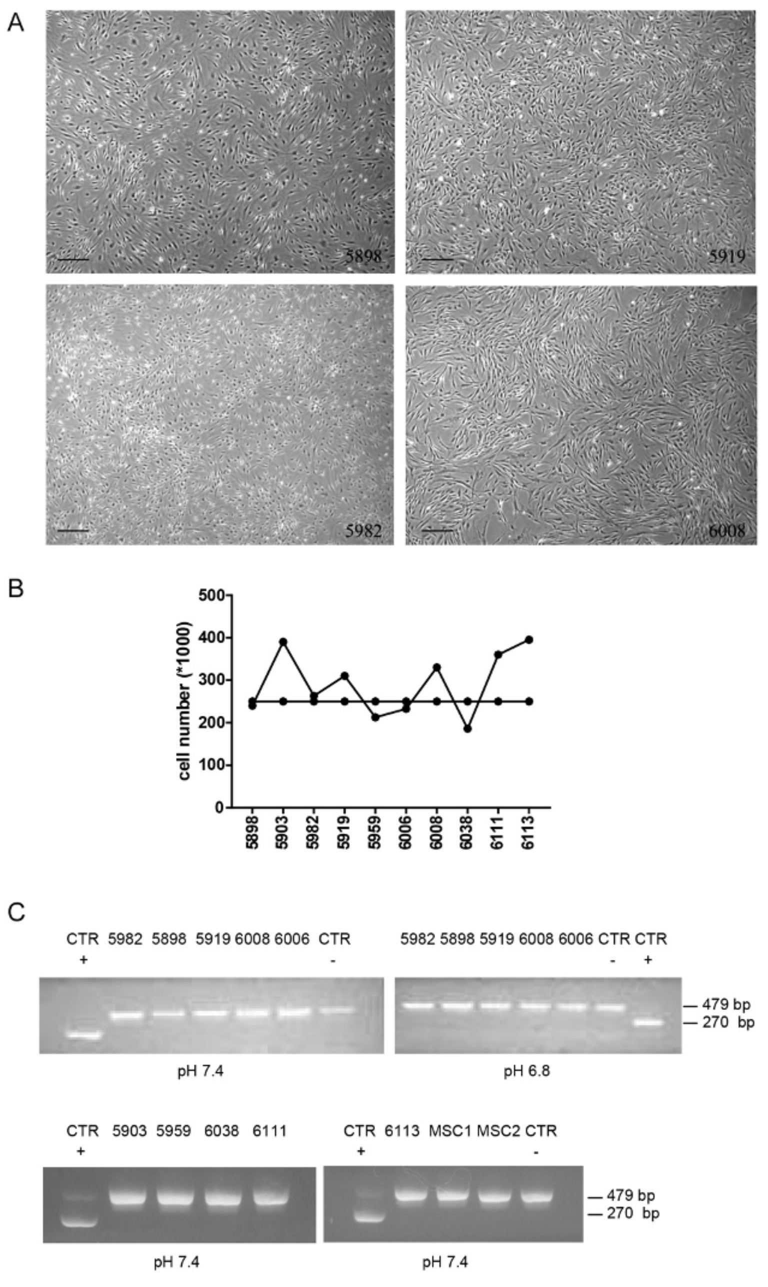


**Supplementary Figure 3.** (A) Representative picture of semi-confluent adMSC samples at passage 3 before conditioned medium harvesting. adMSC showed the typical fibroblast-like morphology. Bars: 50 µm. (B) Cell density in the individual samples, measured in a 25 cm$^2$ flask, at culture supernatant collection time-point. Patient codes are indicated in the x axes. (C) The absence of mycoplasma contamination in the conditioned media harvested from adMSC was verified by a PCR reaction specific for conserved 16S rRNA operon coding region in the Mycoplasma genome. The positive control shows a band at 270 bp and an additional slight band of the internal control at 479 bp. The negative control and negative samples show the internal control band at 479 bp.

Pictures were acquired with bright field microscope (Eclipse TE 2000-S; Nikon; objectives: (Phl 10×/0.25 WD 7.0) coupled with a digital camera (DS-Fi3, resolution 2880 × 2042, 5.9 Mpixel; Nikon), using the NIS-Elements F software (Nikon).

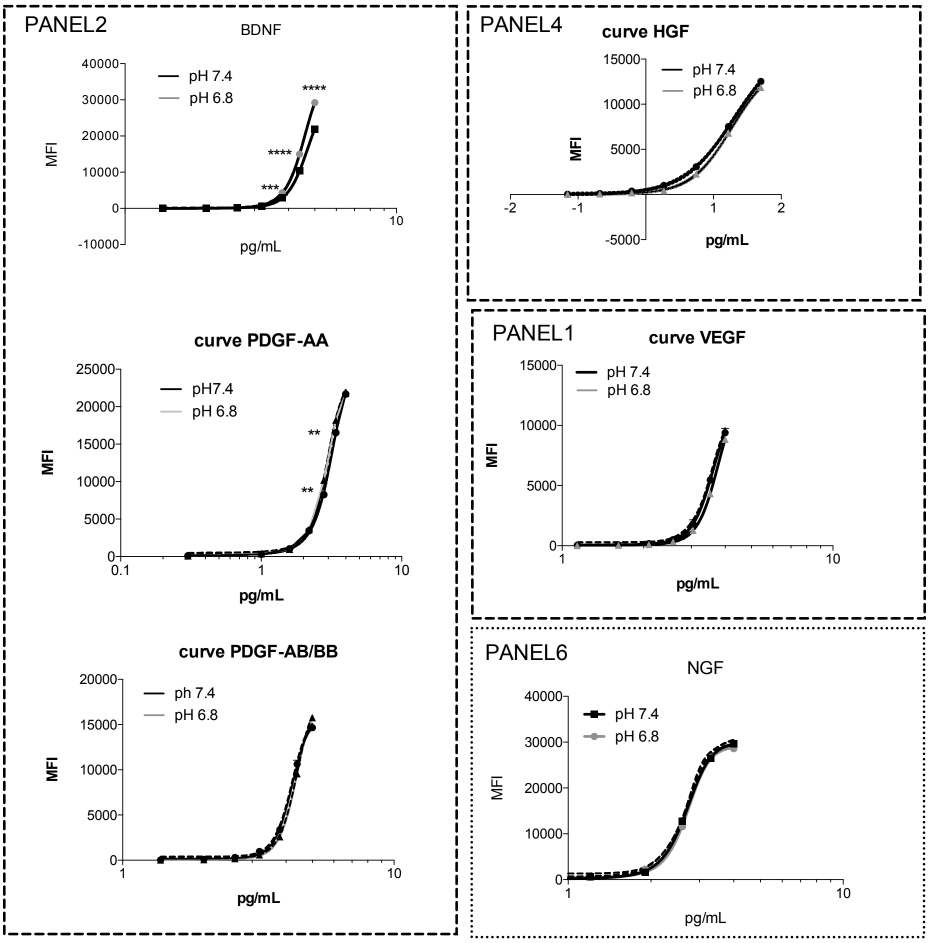


**Supplementary Figure 4.** Effect of low and high pH on standard curves of detectable growth factors. Graphs represent standard curves of detectable analytes obtained adding conditioned medium without cells at different pH. All interpolating curves show $r^2 > 0.98$, indicating no pH effect on assay. Some single standard concentrations show differences depending on pH (indicated with asterisks in the graphs). Each sample has been interpolated on the standard curve added of conditioned medium at the same pH. Statistical analysis. Two-way ANOVA. Asterisks represent the differences between the standard of calibration curves (* $p < 0.05$; ** $p < 0.01$; *** $p < 0.001$; **** $p < 0.0001$).
