# Supplementary material for: Intra-individual variability in the neuroprotective and promyelinating properties of conditioned culture medium obtained from human adipose mesenchymal stromal cells
Source: Stem Cell Res Ther. 2023 May 11;14:128. doi: 10.1186/s13287-023-03344-1 (PMC10173531; doi:10.1186/s13287-023-03344-1)
Supplement: Supplementary file 2 — Additional file 2. Original pictures of the PCR gels for the mycoplasma contamination test showed in Supplementary Figure 3C. [file 13287_2023_3344_MOESM2_ESM.pdf]

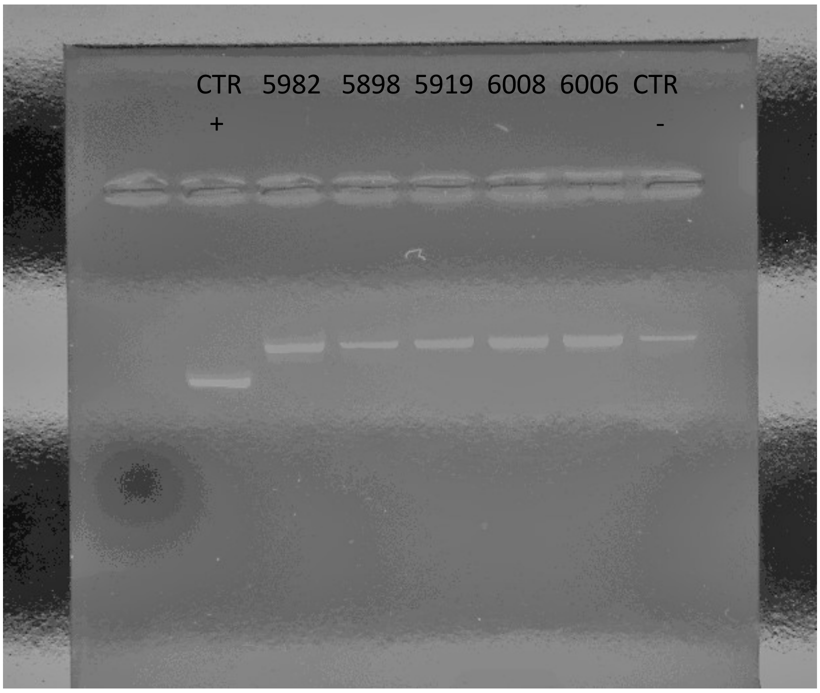

Original pictures (Fig Supplementary 3 first lane pH 7.4).

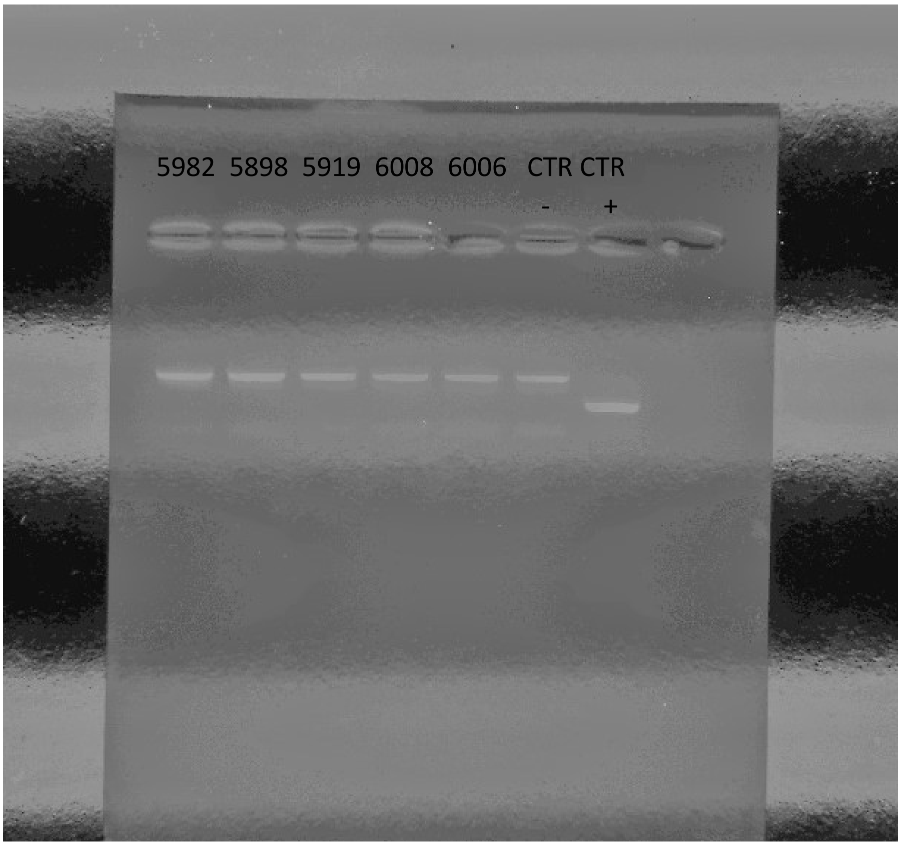

Original pictures (Fig Supplementary 3 first lane pH 6.8).

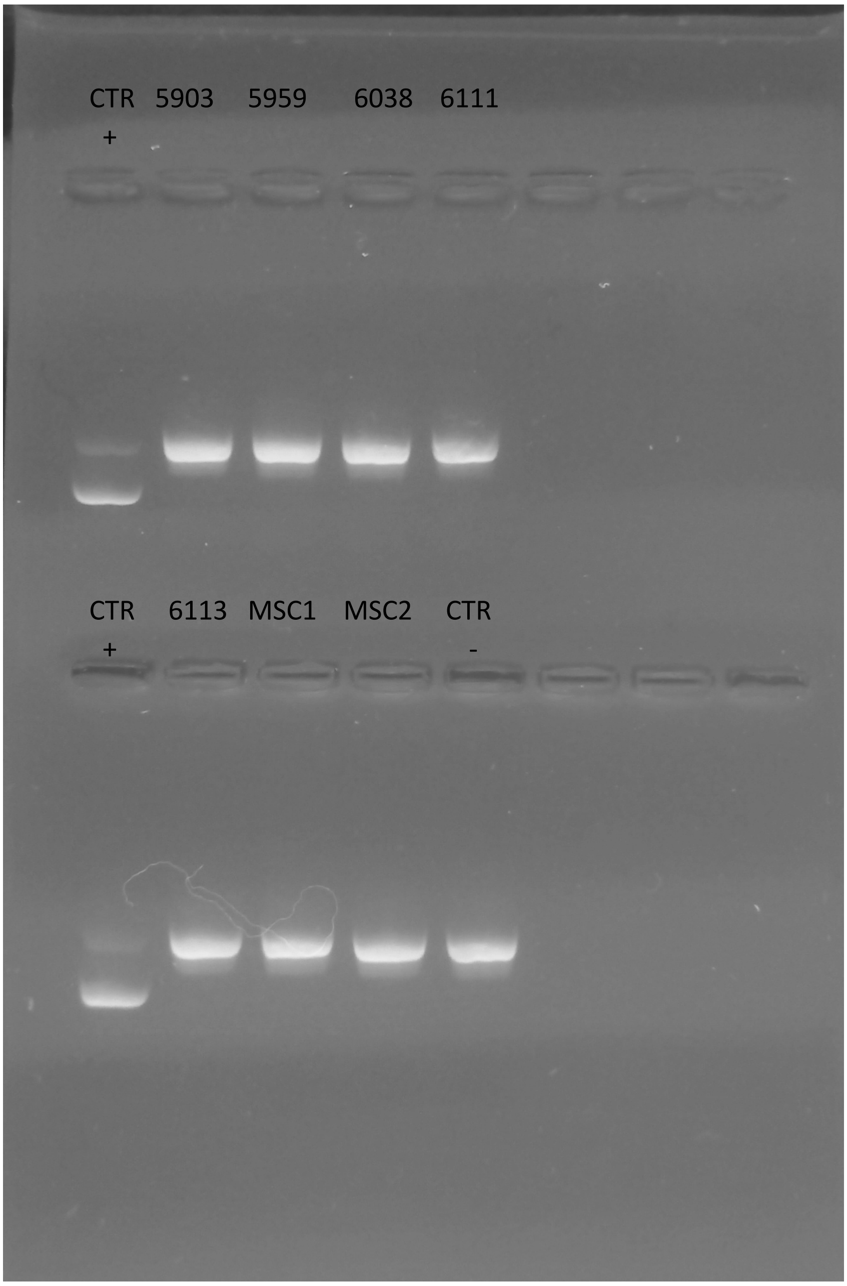

Original pictures (Fig Supplementary 3 second lane pH 7.4 and pH 7.4).
