# Supplementary material for: Intra-individual variability in the neuroprotective and promyelinating properties of conditioned culture medium obtained from human adipose mesenchymal stromal cells
Source: Stem Cell Res Ther. 2023 May 11;14:128. doi: 10.1186/s13287-023-03344-1 (PMC10173531; doi:10.1186/s13287-023-03344-1)
Supplement: Supplementary file 3 — Additional file 3. Table showing the levels of detectable growth factors in 72-hour conditioned media of adMSC. [file 13287_2023_3344_MOESM3_ESM.pdf]

Subject ID	BDNF (pg/10^6 cells)			PDGF-AA (pg/10^6 cells)			PDGF-AB/BB (pg/10^6 cells)			NGF (pg/10^6 cells)			VEGF-A (pg/10^6 cells)			HGF (pg/10^6 cells)		
	av ± SD			av ± SD			av ± SD			av ± SD			av ± SD			av ± SD		
5898	26,37	±	3,92	24,58	±	6,02	59,05	±	0,00	46,88	±	1,47	17037,93	±	511,45	11496,92	±	134,01
5903	42,89	±	2,32	26,88	±	2,29	100,03	±	13,16	30,13	±	2,72	8174,79	±	704,82	2152,71	±	109,11
5919	28,91	±	8,98	2,81	±	3,05	23,87	±	0,00	64,52	±	4,56	8148,62	±	1124,60	4295,58	±	484,05
5959	96,67	±	4,21	27,81	±	2,26	50,75	±	22,53	75,29	±	3,33	10531,16	±	427,35	2000,81	±	9,52
5982	24,06	±	3,58	20,53	±	0,93	28,13	±	0,00	40,87	±	1,34	12857,29	±	377,23	2826,84	±	103,80
6006	315,04	±	3,65	32,62	±	2,00	106,09	±	21,54	104,30	±	7,60	23113,92	±	2677,11	2528,42	±	136,31
6008	126,40	±	2,63	29,61	±	1,71	96,35	±	15,38	100,00	±	32,14	15139,74	±	434,76	1109,72	±	24,78
6038	258,22	±	15,08	45,39	±	1,29	574,73	±	0,00	94,09	±	0,00	16225,12	±	400,47	1362,05	±	0,00
6111	129,48	±	6,68	127,59	±	0,36	148,26	±	54,63	45,14	±	4,91	48250,53	±	1080,98	1183,35	±	20,06
6113	60,68	±	2,02	26,64	±	0,46	99,92	±	0,00	62,03	±	8,95	10281,15	±	629,91	803,11	±	0,00
COMM1	753,83	±	23,62	44,83	±	1,46	191,25	±	11,38	183,62	±	23,16	33937,16	±	1701,15	1327,31	±	75,49
COMM2	1263,26	±	109,72	136,97	±	10,88	212,62	±	26,25	108,00	±	16,97	52330,24	±	1707,13	1846,37	±	57,30

Levels of detectable growth factors in 72-hour conditioned media of adMSC. Data are expressed as mean ± SD and normalized on number of cells at the time of harvest. FGF-21, GDNF, IGF-I, IGF-II, FGF-2 and EGF were undetectable in all media tested.
